# Pressure-specific and multiple pressure response of fish assemblages in European running waters^☆^

**DOI:** 10.1016/j.limno.2013.05.008

**Published:** 2013-09

**Authors:** Rafaela Schinegger, Clemens Trautwein, Stefan Schmutz

**Affiliations:** Institute of Hydrobiology and Aquatic Ecosystem Management, Department of Water, Atmosphere and Environment, BOKU, University of Natural Resources and Life Sciences, Vienna, Austria

**Keywords:** Fish metrics, Water Framework Directive, Multiple pressures, River types

## Abstract

We classified homogenous river types across Europe and searched for fish metrics qualified to show responses to specific pressures (hydromorphological pressures or water quality pressures) vs. multiple pressures in these river types. We analysed fish taxa lists from 3105 sites in 16 ecoregions and 14 countries. Sites were pre-classified for 15 selected pressures to separate unimpacted from impacted sites. Hierarchical cluster analysis was used to split unimpacted sites into four homogenous river types based on species composition and geographical location. Classification trees were employed to predict associated river types for impacted sites with four environmental variables. We defined a set of 129 candidate fish metrics to select the best reacting metrics for each river type. The candidate metrics represented tolerances/intolerances of species associated with six metric types: habitat, migration, water quality sensitivity, reproduction, trophic level and biodiversity. The results showed that 17 uncorrelated metrics reacted to pressures in the four river types. Metrics responded specifically to water quality pressures and hydromorphological pressures in three river types and to multiple pressures in all river types. Four metrics associated with water quality sensitivity showed a significant reaction in up to three river types, whereas 13 metrics were specific to individual river types. Our results contribute to the better understanding of fish assemblage response to human pressures at a pan-European scale. The results are especially important for European river management and restoration, as it is necessary to uncover underlying processes and effects of human pressures on aquatic communities.

## Introduction

The development of fish-based methods for the assessment of human pressures on the aquatic ecosystem has a long history. There has been considerable scientific effort to define appropriate fish metrics and fish indices for the assessment of the ecological status of different types of running waters in the United States (Fausch et al., 1990, Lyons, 1992, McCormick et al., 2001, Mebane et al., 2003, Hughes et al., 2004, Whittier et al., 2007, Pont et al., 2009). Most of the work has been within the framework of the “Clean Water Act”, based on the “Index of Biotic Integrity” (IBI) and the related findings of Karr (1981).

In Europe, the EU Water Framework Directive (WFD, European Commission, 2000) has been a major driver in the development of standardised fish based assessment methods and metrics to determine the ecological status of European rivers and the classification of human degradation (Oberdorff et al., 2001, Oberdorff et al., 2002, Pont et al., 2005, Pont et al., 2007, Roset et al., 2007, Segurado et al., 2011, Logez and Pont, 2011).

Subsequent, EU-funded projects such as FAME (FAME Consortium, 2004) and “European Fish Index Plus (EFI+)” (EFI+ Consortium, 2009), have developed multi-metric indices based on fish assemblages and analysed relationships between fishes and human pressures. Additional studies by Noble et al. (2007b), Melcher et al. (2007), Schmutz et al. (2007b), Virbickas and Kesminas (2007), Grenouillet et al. (2007) and Ferreira et al. (2007) aimed to find appropriate metrics that showed different reactions under unimpacted/impacted conditions for various regions in Europe.

Numerous studies have analysed fish metrics to detect pressures by differentiating between reference and degraded sites (Bailey et al., 1998, Hughes et al., 1998, Karr and Chu, 2000, Hering et al., 2006, Pont et al., 2006, Pont et al., 2009, Stoddard et al., 2008, Southerland et al., 2007, Logez and Pont, 2011). Low quality data and information gaps regarding pressures have produced errors and bias in fish metric responses to different types of pressures. Consequently, although providing reliable results at the large scale, pan-European fish metrics were unable to differentiate between unimpacted and impacted conditions in specific areas, river types or pressure situations (Melcher et al., 2007, Schmutz et al., 2007a, Schmutz et al., 2007b, Pont et al., 2007). The EFI+ project (EFI+ Consortium, 2009, http://efi-plus.boku.ac.at/), tried to overcome these problems by identifying and collecting important pressures across Europe on a more accurate and standardised basis. Based on these data, Schinegger et al. (2012) showed that (1) degradation of European rivers is widespread, (2) single water quality pressures (W) are not dominant, but (3) many European rivers are affected by hydromorphological pressures (HMC) or a combination of pressure types (W + HMC). Furthermore, Schinegger et al. (2012) found that hydromorphological pressures (HMC) are the key pressures in alpine regions and headwaters and water quality pressures (W) and multiple pressures (W + HMC) prevail in lowlands.

According to Hering et al. (2006) and Logez and Pont (2011), the signal reflected by metrics should only display the variability of pressures between sites and not the environmental differences between them. Furthermore, Hughes and Oberdorff (1999), Roset et al. (2007) and Pont et al. (2009) stated that the creation of new IBIs and IBI scoring criteria to suit natural regional and local differences might be unsuitable when applied to areas outside those for which they were developed. Subsequently many studies have focused on a predefined ecoregion approach. The Illies ecoregion system (Illies, 1978) is the only widely used pan-European classification and was adopted by the WFD. However, Schmutz et al. (2007a) argue that the Illies system has never been evaluated for its ability to discriminate among fish assemblages at a continental scale. Schmutz et al. (2007a) also stated that two spatial dimensions structure fish assemblages at the large scale: the zoogeography across Europe and the longitudinal pattern within each river. Schmutz et al. (2007b) and Melcher et al. (2007) then developed the Fish Assemblage Types (FATs) as an underlying concept for a “Spatially Based Method (SBM)” of classification, which divides rivers into units with homogenous fish assemblages (i.e. a river type specific approach). The SBM approach was initially applied to individual ecoregions (Ferreira et al., 2007, Grenouillet et al., 2007, Noble et al., 2007b, Virbickas and Kesminas, 2007), and then simultaneously to all ecoregions (Melcher et al., 2007, Schmutz et al., 2007a). However, as the SBM approach only applies to rivers belonging to FATs defined in previous studies, it is necessary to extend the geographic range of the SBM.

Based on these previous findings, our study represents a pan-European approach to test the response of fish assemblages to pressures in different river types. Our intent was (1) to define homogenous river types across Europe and (2) to find appropriate fish metrics for these types, showing a response to specific and multiple human pressures.

## Methods and data

### Allocation and pre-classification of sites

All data were extracted from an extensive database (EFI+ Consortium, 2007) containing fish surveys conducted by several academic institutions and environmental agencies across Europe. Sites were sampled by electrofishing (wading) during low flow periods using European standards (CEN, 2003). We included only sites with fished areas greater than 100 m^2^ and having more than 50 caught individuals to minimise the risk of false absences.

Due to multiple sampling sites located in one river, we applied another selection step to compensate for possible spatial autocorrelation. Dispersed distribution of sampling sites was defined in three classes based on upstream catchment size and three thresholds for distance along the stream network between sampling sites. Threshold for (1) small catchments (<1000 km^2^) was >5 km distance, (2) for medium catchments (1000–10,000 km^2^) >10 km, and (3) for large catchments (>=10,000 km^2^) >50 km. The dataset comes for sites from 2079 rivers of which 1553 (74.6%) rivers are associated with only one sampling site, 307 (14.8%) rivers are associated with two sampling sites, and 218 (10.5%) rivers are associated with three or more sampling sites within the entire river. Median catchment size is 82 km^2^ and 90% of the sites have a catchment size below 1000 km^2^.

After this first step, 3105 sites in 16 ecoregions and 14 countries were available for our analyses. Pre-classification of sites was done for 15 selected pressure variables (Table 1) in order to separate unimpacted sites (no or very slight pressure) from strongly impacted sites. Pressure variables were selected by Schinegger et al. (2012) according to known effects on aquatic habitats and organisms.

**Table 1:** Selected pressures for unimpacted/impacted site differentiation. Type indicates if the pressure is considered as water quality pressure (W) or hydromorphological pressure including connectivity (HMC, for details see Schinegger et al. 2012).

Pressure variable	Type	Explanation; short description of classes
Impoundment	HMC	Natural flow velocity reduction on site due to impoundment; 1 = no (no impoundment), 3 = weak, 5 = strong;
Hydropeaking	HMC	Site affected by hydropeaking; 1 = no (no hydropeaking), 3 = partial, 3 = yes;
Water abstraction	HMC	Site affected by water flow alteration/minimum flow; 1 = no (no water abstraction), 3 = weak to medium (less than half of the mean annual flow), 5 = strong (more than half of mean annual flow);
Reservoir flushing	HMC	Fish fauna affected by flushing of reservoirs upstream of site; 1 = no, 3 = yes;
Hydrograph modification	HMC	Seasonal hydrograph modification due to hydrological alteration (water storage for irrigation, hydropower etc.); 1 = no, 3 = yes;
Channelisation	HMC	Alteration of natural morphological channel plan form; 1 = no, 3 = intermediate, 5 = straightened;
Cross section alteration	HMC	Alteration of cross section; 1 = no, 3 = intermediate, 5 = technical cross section./U-profile
Instream habitat alteration	HMC	Alteration of instream habitat conditions; 1 = no, 3 = intermediate, 5 = high;
Embankment	HMC	Artificial embankment; 1 = no (natural shoreline), 2 = slight (local presence of artificial material for embankment), 3 = intermediate (continuous embankment but permeable), 5 = high (continuous, no permeability);
Flood protection	HMC	Presence of dykes for flood protection; 1 = no, 3 = yes;
Barriers segment upstream	HMC	Barriers on segment level upstream; 1 = no, 3 = partial, 3 = yes^a^;
Barriers segment downstream	HMC	Barriers on segment level downstream; 1 = no, 4 = partial, 4 = yes^a^;
Acidification	W	Acidification; 1 = no, 3 = yes;
Eutrophication	W	Artificial eutrophication; 1 = no, 3 = low, 4 = intermediate (occurrence of green algae), 5 = extreme (oxygen depletion);
Organic pollution	W	Is organic pollution observed; 1 = no, 3 = weak, 5 = strong;

a Partial barriers and yes are considered to have the same impact.

**Fig. 1:**
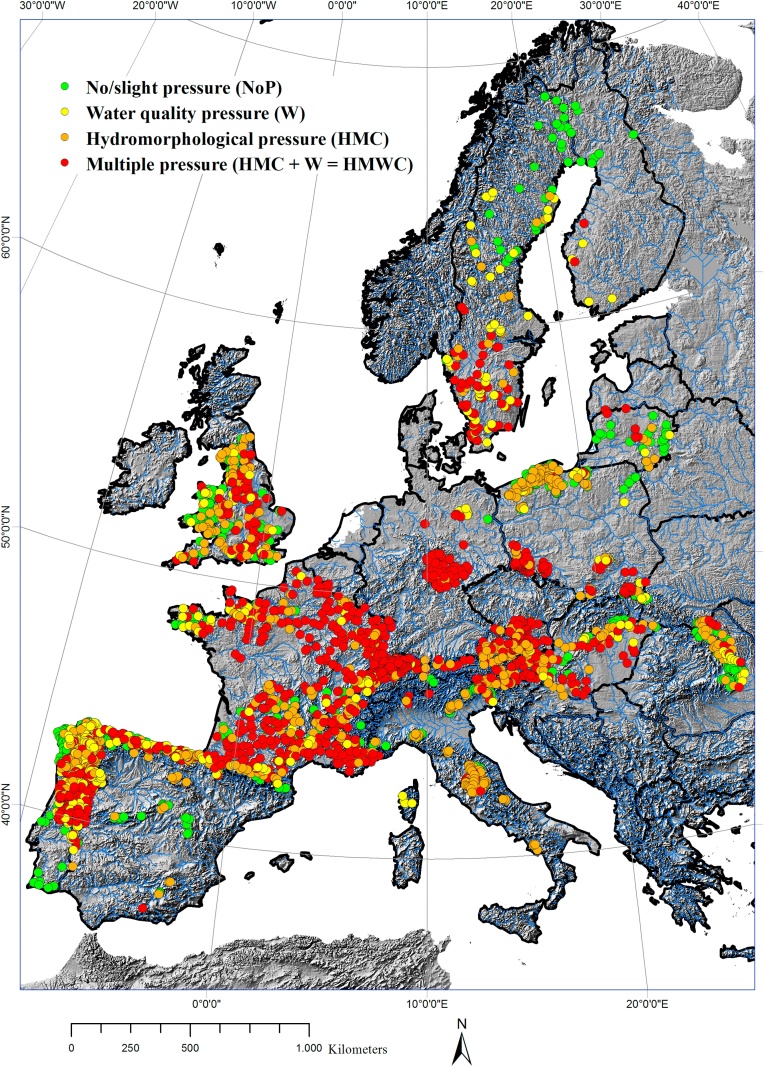
Spatial location and pressure status of sites [*n* = 3105].

In total, 716 sites were classified as unimpacted (classes 1 and 2) and 2389 sites as impacted (classes 3, 4 and 5). Furthermore, impacted sites were associated with specific pressures and pressure combinations according to Schinegger et al. (2012), (see “Group” in Table 1 for details). In this context, 390 sites were impacted only by water quality pressures (W), 771 sites only by hydromorphological pressures (HMC) and 1228 sites by multiple pressures (HMWC), i.e. a combination of water quality and hydromorphological pressures (Schinegger et al., 2012). Fig. 1 shows the spatial location and pressure status of sites.

### Fish metrics description

As suggested the EFI+ Consortium (2009), six structural and functional types of metrics were considered for candidate metrics: biodiversity, habitat, migration, reproduction, trophic level and water quality sensitivity. In the dataset, 116 fish species were assigned to tolerances related to these attributes according to the EFI+ classification, based on previous literature and completed by expert judgement (Holzer, 2008, EFI+ Consortium, 2009; Annex Table 1).

In total, 129 candidate metrics were pre-selected for further analyses (Table 2). The selected metrics included six variants: number of species, density (number of individuals per ha) and biomass (kg per ha) per metric as well as relative information on number of species, density and biomass (as percentage of total species). According to Noble et al. (2007a) and Virbickas and Kesminas (2007), these variants reflect most of the important ecological aspects of metrics. Associated references and reactions can be found in Table 2. As information on fish length was not available for a large part of our dataset, we decided not to consider metrics based on size classes/life stages.

**Table 2:** Name and definition of candidate metrics for further analyses. Type: biodiv = biodiversity, hab = habitat, mig = migration, repro = reproduction, troph = trophic level, wq = water quality; Variants: nsp = number of species, dens = density [Ind/ha], biom = biomass [kg/ha], perc_nsp: number of species of guild in relation to all species, perc_dens = density of guild in relation to all guilds, perc_biom = biomass of guild in relation to all guilds, all = all six variants are included; Direction: incr = metrics that increases with increasing human pressure, decr = metric that decreases with increasing human pressure; Reaction according to reference in text.

Metric name	Definition	Type	Variants	Direction	Reaction
Nsp_all	Total number of fish species, including native and alien species.	biodiv	nsp, dens, biom	decr/incr	Generally inclines along the longitudinal course of a river, increase in species poor river types and decrease in lowland rivers considered as pressure.
Nsp_native	Number of native species.	biodiv	nsp, dens, biom, perc_nsp	decr	Native species.
Nsp_alien	Number of alien species.	biodiv	dens, biom	incr	Replace native species.
HTOL_HINTOL	Habitat degradation intolerance.	hab	all	decr	Reaction of species with narrow habitat flexibility.
HTOL_HTOL	Habitat degradation tolerance.	hab	all	incr	Reaction of species having a large flexibility in terms of habitat degradation.
Hab_EURY	Degree of rheophily. Fish that exhibit a wide tolerance of flow conditions, although generally not considered to be rheophilic.	hab	nsp, dens, perc_nsp, perc_dens	incr	Degradation of lotic and lentic habitats.
Hab_LIMNO	Degree of rheophily. Fish prefer to live, feed and reproduce in a habitat with slow flowing to stagnant conditions.	hab	all	incr	Degradation of lentic habitats.
Hab_RH	Degree of rheophily. Fish prefer to live in a habitat with high flow conditions and clear water.	hab	all	decr	Degradation of lotic habitats.
HabSp_LIPAR	Preference to spawn in stagnant waters.	hab	all	incr	Degradation of lentic spawning habitats.
HabSp_RHPAR	Preference to spawn in running waters.	hab	all	decr	Degradation of lotic spawning habitats.
Mig_POTAD	Species migrate between river zones or more than 5–10 km.	mig	all	decr	Sensitive to ecologicial connectivity of river systems.
Repro_PELA	Fish spawn into the pelagic zone.	repro	all	incr	Degradation of pelagic spawning habitats.
Repro_PHYT	Fish deposit eggs in clear water habitats on submerged plants.	repro	all	decr	Degradation of plant-related spawning habitats.
Repro_POLY	Non-specialised spawners.	repro	all	incr	Degradation of spawning habitats.
Repro_LITH	Fish spawn exclusively on gravel, rocks, stones, rubbles or pebbles, hatchlings are photophobic.	repro	all	decr	Degradation of gravel spawning habitats, sensitive to siltation.
Atroph_INSV	Insectivorous species.	troph	all	decr	Surrogate for evaluating the degree that the invertebrate assemblage is degraded by human pressures.
Atroph_PISC	Piscivorous species.	troph	all	decr	Top predator, surrogate for prey fishes.
Atroph_PLAN	Planktivorous species.	troph	all	decr	Surrogate for plankton.
Atroph_OMNI	Food of adult consists of more than 25% plant material and more than 25% animal material. Generalists.	troph	all	incr	Degree that the food base is altered to favour species that can digest both plant and animal foods.
WQgen_INTOL	In general intolerant to usual water quality parameters.	wq	all	decr	Reaction of species with narrow flexibility in terms of water quality degradation.
WQgen_TOL	In general tolerant to usual water quality parameters.	wq	all	incr	Reaction of species having a wide flexibility in terms of water quality degradation.
WQO2_O2INTOL	Tolerant to low Oxygen concentration. More than 6 mg/l in water.	wq	all	decr	Reaction of species with narrow flexibility in terms of oxygen concentration problems.
WQO2_O2TOL	Tolerant to low Oxygen concentration: 3 mg/l or less.	wq	all	incr	Reaction of species having a wide flexibility in terms of oxygen concentration problems.

### River type modelling

To classify fish data in similar groups across Europe, homogenous river types (river types) based on fish assemblage data were modelled using only unimpacted sites. We conducted a hierarchical cluster analysis (agnes, R Core Development Team, 2011) after Ward's method, with Euclidean distance as similarity measure including four fish metrics: percentage of lithophilic species (Repro_LITH_perc_nsp), percentage of omnivorous species (Atroph_OMNI_perc_nsp), percentage of potamodromous species (Mig_POTAD_perc_nsp) and percentage of rheophilic species (Hab_RH_perc_nsp) as well as geographic position to include regionalisation. The threshold for identifying distinct river types was set by eye in the cluster dendrogram to find a feasible number of strong and well-separated river types.

According to Hughes et al. (2004), Whittier et al. (2006) and Pont et al. (2009), lotic fish assemblages are limited by many natural variables as elevation, temperature, precipitation, flow regime, and channel slope etc. Therefore, to describe the local environmental characteristics of the sampling sites, we conducted classification tree analysis (rpart, R Core Development Team, 2011) with river type as dependent variable and seven environmental variables as possible descriptors: altitude, river slope, mean annual precipitation, mean annual air temperature, mean air temperature in January, latitude and longitude. These variables were chosen because they describe both the regional position in the hydrographic network and the organisation of sites along the longitudinal continuum of rivers. River slope is the drop of altitude divided by river segment length [m/km], where segment length is 1 km for small streams (<100 km^2^), 5 km for intermediate rivers (100–1000 km^2^) and 10 km for large rivers (>1000 km^2^). River slope was measured in maps with scale 1:50,000 or 1:100,000.

The chosen model fitting algorithm ‘rpart’ uses a 10-fold cross-validation. The training set is split into 10 roughly equally sized parts and the tree is grown on nine parts while using the tenth for testing (Venables and Ripley, 2003). The results are averaged and expressed as xerror, which is the cross-validated error estimation of the model as mean square error of the predictions at each split in the tree. Only four of the seven environmental variables (altitude, mean annual air temperature, mean annual precipitation and latitude) were finally used by the algorithm for tree construction.

Next, a prognosis of river types for impacted sampling sites based on the ‘rpart’ model was conducted, i.e. river type affiliation for impacted sites was modelled based on the four environmental variables. By comparing the mean metric values of unimpacted with impacted sites within each river type we can define the river type specific sensitivity and intensity of the alteration of fish assemblages as a reaction to human pressures. To avoid extrapolation in the prediction, impacted sites outside the range of the environmental characteristics of the unimpacted sites (between 5% and the 95% percentile) were eliminated from the dataset.

### Response of metrics to pressures

As some fish metrics decrease in response to increasing human pressures (less fish of a guild leading to reduced density and biomass, disappearance of species) but in contrast, several others tend to increase (e.g. metrics associated with generalist and tolerant species), their testing for sensitivity and intensity is in reverse direction. Therefore, we set the direction of a metric's response to human pressures from literature (Oberdorff, 1996, Oberdorff et al., 2002, Karr, 1981, Verneaux, 1981, Grandmottet, 1983, Pont et al., 2006, Melcher et al., 2007, Noble et al., 2007a, Logez and Pont, 2011) and later used this classification for the direction of statistical tests (Table 2). Out of a total of 129 metrics, we defined 79 metrics as decreasing with human pressure, 49 as increasing and one metric as both, increasing and decreasing (*Nsp_all*, including both native and non-native species, see Table 2).

A one-sided Welch two sample *t*-test (Bonferroni-correction, *p* = 0.083) was used to test the differences between mean metric values of unimpacted and impacted sites (sensitivity), estimating *p*-values with the alternative hypothesis that the true difference in means (unimpacted–impacted) is greater than zero for those metrics supposed to decrease with increasing human pressure. Metrics classified as increasing are tested with the alternative hypothesis that the true difference in means is less than zero.

Furthermore, we used the ecological quality ratio (EQR) to identify the intensity of metric response between unimpacted and impacted conditions.

For metrics classified as decreasing with increasing pressure, the EQR was calculated as follows:

$${\text{eqr}}_{\text{RT}i}=\frac{\bar{x}[{\text{impacted}}_{\text{RT}i}]}{\bar{x}[{\text{unimpacted}}_{\text{RT}i}]}$$

where *i* = 1…4, *x* is the arithmetic mean of fish metric values and RT the river type.

The EQR is calculated inverse for metrics classified as increasing with increasing pressure:

$${\text{eqr}}_{\text{RT}i}=\frac{\bar{x}[{\text{unimpacted}}_{\text{RT}i}]}{\bar{x}[{\text{impacted}}_{\text{RT}i}]}$$

where i = 1…4, *x* is the arithmetic mean of fish metric values and RT the river type.

This is to ensure an EQR scale from 0 (impacted) to 1 (unimpacted condition). A metric was classified as qualified if it showed a significant difference between unimpacted vs. impacted sites (*p* < 0.05) and if the EQR was less than 0.7 – i.e. if the difference between unimpacted and impacted condition was greater than 30%. Furthermore, we tried to avoid biased results due to high frequencies of zero values for specific metrics in certain river types. Frequent true zero values can occur for metrics related to rare species (e.g. piscivorous guild) if absent in reference conditions and, hence, cannot decrease in impacted conditions. Therefore, we defined that at least 50% of sites must have a valid metric value (unequal to zero, i.e. for metrics classified as decreasing >50% of unimpacted sites, for sites classified as increasing >50% of impacted sites). The metric response tests for sensitivity and intensity were conducted within each river type separately.

To avoid redundancy, Pearson correlation analysis was conducted for the overall dataset and for each river type separately for the final selection, i.e. for metrics with a correlation coefficient higher or equal 0.7, only one (the first) metric was retained for the final metric list.

To prove if the selected metrics respond to specific pressures or pressure combinations (multiple pressures), paired *t*-tests were then repeated to show the response between unimpacted sites (NoP), sites impacted only by hydromorphological pressures (HMC), sites impacted only by water quality pressure (W), or by multiple pressures (HMCW). In the results section, this difference is figured out with notched boxplots: if the notches do not overlap, this is strong evidence that their medians differ (Chambers et al., 1983, p. 62). All statistical analyses were performed in R version 2.13.1 (R Core Development Team, 2011).

## Results

### Metrics selection

Restrictions for the occurrence of zero-values in the data led to the exclusion of 54 of the 129 candidate metrics. In total, 31 metrics showed a significant response to human pressures (unimpacted vs. impacted) and a high EQR (> than 30% change). Furthermore, 14 metrics were removed in the next step due to redundancy based on numerous correlations with other metrics (correlation coefficient >0.7; see Annex Table 2, Annex Table 3, Annex Table 4, Annex Table 5 for details). Finally, 17 metrics were selected for final testing of pressure specific and multiple pressure responses.

### River types

The river types were defined based on fish community; the environmental characteristics were associated lately with the classification tree. This resulted in four river types. The classification tree model could classify 75% of the 716 overall reference sites correctly. The correct ratio for HWS was 63%, for MGR 81%, for LLR 77% and for MES 80%. The validation of the model supports a quite stable model with an estimated error of 0.43 rising to 0.52 in tenfold cross-validation. The river types can be classified as follows: head water streams (HWS), medium gradient rivers (MGR), lowland rivers (LLR) and Mediterranean streams (MES, a special type in the Peri-Mediterranean area of Europe according to Reyjol et al., 2007 in which there are many basin-endemic taxa according to Segurado et al., 2011).

HWS are inhabited by 86% lithophilic species, 70% potamodromous, 78% rheophilic, and 6% omnivorous species (Table 3). MGR and MES showed similar means in lithophilic, omnivorous, and potamodromous species but differed in rheophilic species (MGR: 84%, MES: 51%). LLR bore the highest mean of omnivorous (32%) and the smallest of lithophilic species (42%). Furthermore, species composition (based on the total number of individuals caught per species) also showed clear differences (Table 3): HWS were highly dominated by brown trout (*Salmo trutta*), MGR were dominated by European minnow (*Phoxinus phoxinus*) and brown trout. LLR were associated with assemblages dominated by roach (*Rutilus rutilus*) and gudgeon (*Gobio gobio*) and MES were dominated by brown trout, minnow and dace (*Leuciscus souffia*). The environmental characteristics of river types are shown in Table 4.

**Table 3:** Association with percentage of species metrics used for river type modelling and distribution of fish species (based on the total number of individuals caught per species) in modelled river types (HWS = head water streams, MGR = medium gradient rivers, LLR = lowland rivers, MES = Mediterranean streams).

HWS				MGR				LLR				MES			
Metric for RT modelling	% Total	Fish species	% Total catch	Metric for RT modelling	% Total	Fish species	% Total catch	Metric for RT modelling	% Total	Fish species	% Total catch	Metric for RT modelling	% total	Fish species	% total catch
Repro_LITH_perc_nsp	86	Salmo trutta	70.3%	Repro_LITH_perc_nsp	69	Phoxinus phoxinus	25.6%	Repro_LITH_perc_nsp	42	Rutilus rutilus	16.0%	Repro_LITH_perc_nsp	65	Salmo trutta	33.6%
Atroph_OMNI_perc_nsp	6	Pseudochondrostoma duriense	7.6%	Atroph_OMNI_perc_nsp	13	Salmo trutta	20.0%	Atroph_OMNI_perc_nsp	32	Gobio gobio	13.7%	Atroph_OMNI_perc_nsp	17	Phoxinus phoxinus	11.8%
Mig_POTAD_perc_nsp	70	Squalius pyrenaicus	5.6%	Mig_POTAD_perc_nsp	47	Cottus gobio	9.6%	Mig_POTAD_perc_nsp	33	Salmo trutta	12.1%	Mig_POTAD_perc_nsp	43	Leuciscus souffia	7.0%
Hab_RH_perc_nsp	78	Others	16.5%	Hab_RH_perc_nsp	84	Barbatula barbatula	9.3%	Hab_RH_perc_nsp	58	Phoxinus phoxinus	11.0%	Hab_RH_perc_nsp	50	Rutilus rubilio	6.4%
						Salmo salar	7.5%			Alburnoides bipunctatus	8.4%	Anguilla anguilla	6.4%		
						Others	27.9%			Cottus gobio	5.9%			Others	34.8%
		Total number individuals	21,506							Others	33.0%				

		Mean # species	1.66				51,503				24,757				12,538
		SD # species	0.92				4.4				7.23				3.47
							2.6				3.23				1.71

**Table 4:** Median, range and standard deviation (SD) of environmental characteristics for four river types (HWS = head water streams, MGR = medium gradient rivers, LLR = lowland rivers, MES = Mediterranean streams).

River type		Altitude [m.a.s.l.]^a^	Mean annual air temperature [°C]^a^	Mean annual precipitation [mm]^a^	Latitude^a^	Longitude	Mean january air temperature [°C]	Slope [‰]	Catchment size [km^2^]	Distance from source [m]
HWS	Median	478.0	12.3	1189.6	43.03943	−7.327000	6.0	15.9	21.0	8.0
	Range	0.0–2043.0	1.1–14.6	557.8–1564.5	37.84787–58.87025	−9.090744–26.547563	−6.4–9.7	0.9–294.6	1.0–681.0	1.0–62.0
	SD	363.5	2.6	254.4	2.909860	8.563482	4.1	38	79.1	9.4

MGR	Median	210	8.1	763.9	48.43017	15.961294	−3.5	7.1	47.0	13.0
	Range	1.0–1595.0	−2.3–14.7	474.3–1623.3	40.22691–68.49354	−8.552902–29.509454	−15.8–9.7	0–194.5	1.0–40157.0	1.0–521.0
	SD	291.1	3.4	251.7	6.771172	11.789414	5.5	21.1	4158.7	71.8

LLR	Median	75.0	7.7	659.1	53.85842	17.456455	−3.5	1.8	91.0	17.0
	Range	0.0–470.0	2.4–15.9	562.4–1277.9	39.57362–63.66892	−8.995163 27.042127	−10.3–11.2	0–28.8	2.0–6855.0	1.0–240.0
	SD	82.2	2.1	108.7	3.799002	8.992162	3.9	4.4	1159.6	47.7

MES	Median	208.0	13.7	1101.5	42.66090	−7.744205	7.0	12.4	27.0	10.0
	Range	2.0–1275.0	8.9–17.0	522.5 – 1562.0	37.18996–53.99423	−9.245325–13.390564	−10.3–11.2	0.01–97.2	2.0–1163.0	2.0–89.0
	SD	294.0	1.7	280.3	2.142648	6.236943	2.8	18.4	131.2	11.5

a Indicates variables used for river type modelling.

**Fig. 2:**
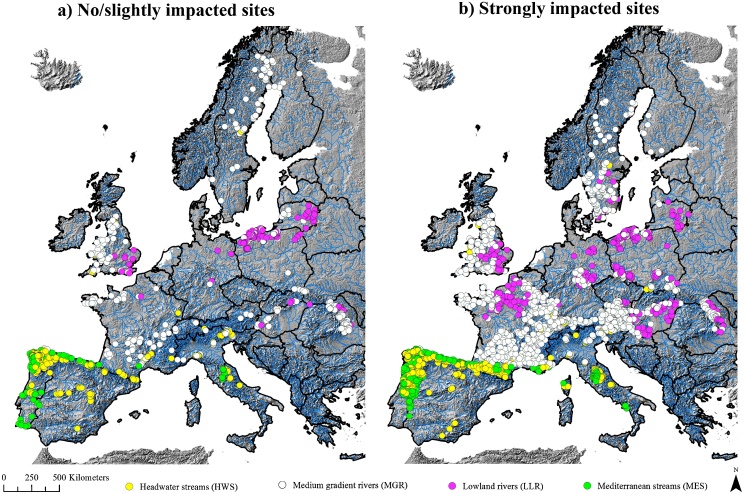
(a) assignment of unimpacted/slightly impacted sites (*n* = 716) to four modelled river types; (b) geographical distribution of strongly impacted sites (*n* = 2389) and modelled association with river types.

In total, 22% of sites (unimpacted and impacted) were located in HWS, 48% in MGR, 15% in LLR and 15% in MES (Fig. 2). Table 5 shows the association of these sites with specific and multiple human pressure status.

**Table 5:** Number (*N*) and percentage (PERC) of sites associated with specific (W, HMC) and multiple (HMWC) human pressures in modelled river types (HWS = head water streams, MGR = medium gradient rivers, LLR = lowland rivers, MES = Mediterranean streams).

	River type									
Pressure type	HWS		MGR		LLR		MES		All sites	
	*N*	PERC	*N*	PERC	*N*	PERC	*N*	PERC	*N*	PERC
NoP	195	28.6%	296	19.7%	128	27.3%	97	21.5%	716	23.1%
HMC	209	30.6%	351	23.4%	102	21.7%	109	24.1%	771	24.8%
W	120	17.6%	151	10.1%	37	7.9%	82	18.1%	390	12.6%
HMWC	159	23.3%	703	46.8%	202	43.1%	164	36.3%	1228	39.5%

Total	683	100.0%	1501	100.0%	469	100.0%	452	100.0%	3105	100.0%

### Pressure specific reaction

One metric responded in river types HWS, LLR and MES: Density of species intolerant to water quality degradation (WQgen_INTOL_dens). Another four metrics responded in these river types: Density of species intolerant to O2 depletion (WQO2_O2INTOL_dens) in HWS and MES; number of species tolerant to water quality degradation (WQgen_TOL_nsp) in MGR and MES; percent density of species intolerant to water quality degradation (WQgen_INTOL_perc_dens) in LLR and MES and percent density of species tolerant to water quality degradation (WQgen_TOL_perc_dens) in MGR and MES. The remaining 12 metrics were specific to individual river types (Table 6).

**Table 6:** Final selection of metrics per river type (HWS = head water streams, MGR = medium gradient rivers, LLR = lowland rivers, MES = Mediterranean streams): Associated metric type (Type) bio = biodiversity, hab = habitat, troph = trophic level, wq = water quality; expected reaction under pressure (Reaction) incr = increasing, decr = decreasing; ** indicates a significant difference between unimpacted and impacted conditions in general (p.gen); significant pressure specific responses (p.spec) related to W = water quality pressure, HMC = hydromorphological pressure including connectivity, HMWC = combination of W + HMC. In bold: metrics reacting either to W or to HMC.

Metric	Type	Reaction	River type							
			HWS		MGR		LLR		MES	
			P.gen	P.spec	P.gen	P.spec	P.gen	P.spec	P.gen	P.spec
WQgen_INTOL_dens	wq	decr	**	HMC, W, HMWC			**	**W**	**	HMC, W, HMWC
WQO2_O2INTOL_biom	wq	decr	**	**W**, HMWC						
HabSp_RHPAR_dens	hab	decr	**	HMC, W, HMWC						
WQO2_O2INTOL_dens	wq	decr	**	HMC, W					**	xxx
WQgen_INTOL_perc_nsp	wq	decr	**	HMC, W, HMWC						
Nsp_all	bio	incr	**	HMC, W, HMWC						
WQgen_TOL_biom	wq	incr					**	HMWC		
HTOL_HTOL_perc_biom	hab	incr					**	HMWC		
WQgen_INTOL_perc_dens	wq	decr					**	**W**, HMWC	**	HMC, W, HMWC
Atroph_PISC_perc_nsp	troph	decr					**	**HMC,** HMWC		
WQO2_O2TOL_perc_nsp	wq	incr					**	HMWC		
Atroph_OMNI_perc_biom	troph	incr					**	HMWC		
WQgen_TOL_perc_dens	wq	incr			**	HMWC			**	**HMC**, HMWC
WQgen_INTOL_biom	wq	decr							**	HMWC
WQgen_TOL_nsp	wq	incr			**	HMWC			**	**HMC**, HMWC
WQO2_O2INTOL_perc_nsp	wq	decr							**	HMWC
Atroph_OMNI_perc_nsp	troph	incr							**	**HMC**, HMWC

**Fig. 3:**
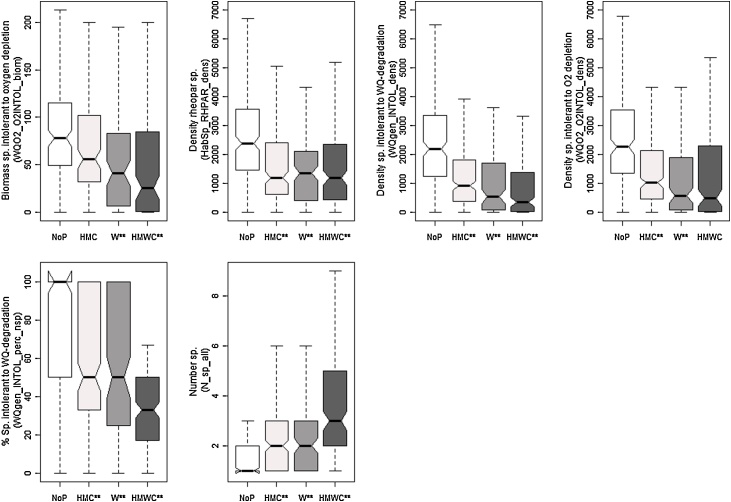
Response of final metric selection for HWS to different pressure types. ** indicates significant difference according to *t*-tests.

**Fig. 4:**
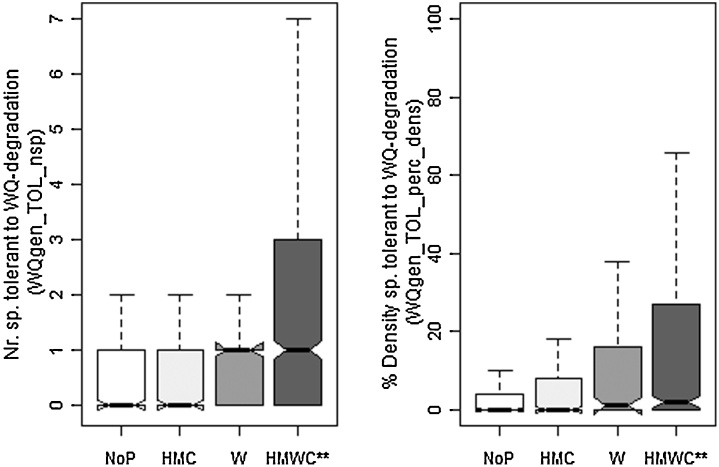
Response of final metric selection for MGR to different pressure types. ** indicates significant difference according to *t*-tests.

For HWS, six metrics finally were selected. All showed significant responses to water quality pressures and five metrics responded to hydromorphological pressure and multiple pressures (Fig. 3). They are associated with water quality sensitivity type (4 metrics), and habitat and biodiversity type (one metric each) (Table 6). For MGR, two metrics finally were selected; both only showed a significant reaction between unimpacted vs. multiple pressures (Fig. 4) and both are associated with water quality sensitivity metric type (Table 6).

**Fig. 5:**
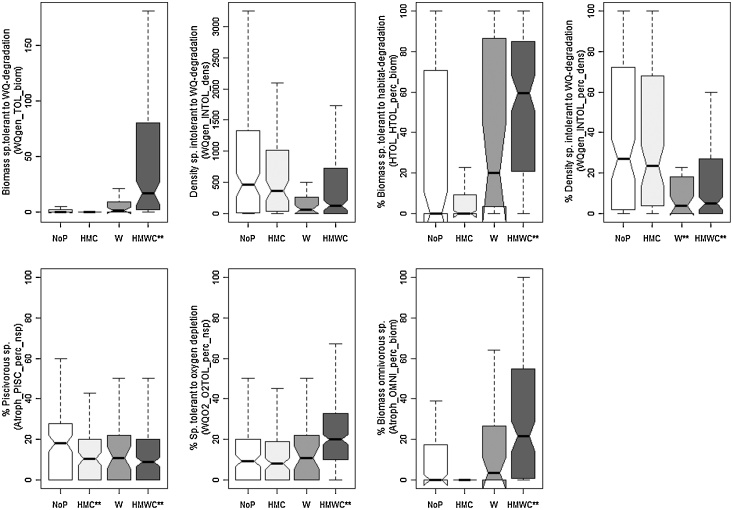
Response of final metric selection for LLR to different pressure types. ** indicates significant difference according to *t*-tests.

For LLR, seven metrics finally were selected. Three metrics showed a response to specific pressures (two metrics to water quality pressures and one to hydromorphological pressures). Six metrics showed a significant response only to multiple pressures (Fig. 5). One metric is associated with habitat-, two with trophic, and four with water quality sensitivity metric group (Table 6).

**Fig. 6:**
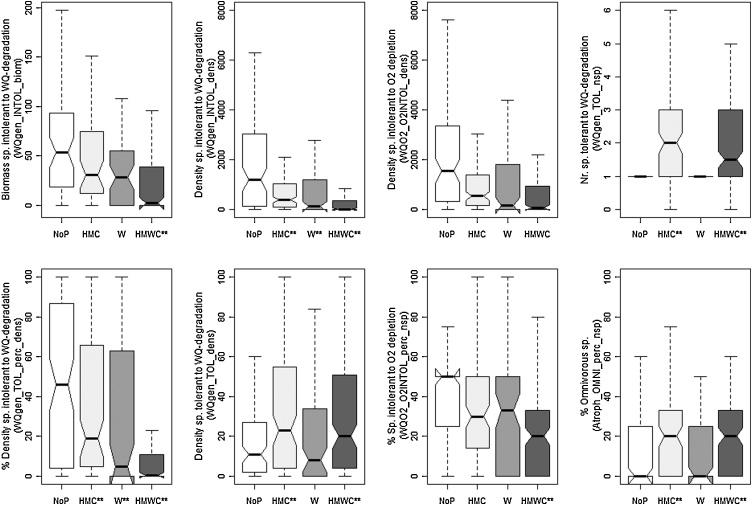
Response of selected metric selection for MES to different pressure types. ** indicates significant difference according to *t*-tests.

For MES, eight metrics finally were selected. Five metrics showed a significant response to hydromorphological pressures, two metrics to water quality pressures and seven metrics to multiple pressures. One metric (WQO2_O2INTOL_dens) showed no significant pressure specific and multiple pressure response (Fig. 6). Seven metrics are associated with water quality sensitivity metric type, only one with trophic level metric type (Table 6).

Overall, eight of the 17 finally selected metrics were “absolute number” metrics (three “biomass” metrics, three “density metrics” and two “number of species” metrics). The other nine metrics were “relative” metrics (five “percentage of species”, two “percentage of density” and two “percentage of biomass”).

Overall, eight metrics were sensitive only to multiple pressures (HMCW), however we found seven “diagnostic metrics”, that reacted exclusively to water quality pressures (Table 6, in bold). Further three metrics showed a response to both specific pressures (W and HMC) and multiple pressures (Table 6) and one metric only to specific pressures.

## Discussion

Many attempts were already made to identify metrics that show a response to human pressures and to generate multi-metric (fish) indices for the evaluation of the ecological status of running waters all over the globe (e.g. Karr, 1981, Fausch et al., 1990, Hughes et al., 2004, Oberdorff and Hughes, 1992, Pont et al., 2009). Moreover, the FAME Consortium (2004), Pont et al. (2007) and the EFI+ Consortium (2009) had already developed fish-based assessment methods derived from very large datasets across Europe. However, for the development of these methods the response of metrics to specific human pressures, i.e. hydromorphological-, water quality- and multiple pressures in different river types were not tested before.

### River types

We classified four homogenous river types across Europe based on the percentage composition of rheophilic, lithophilic, omnivorous and potamodromous species. These metrics were selected based on the assumption that they gave a representative overview of the dominating fish assemblages in our dataset. Fish Assemblage Types (FATs) were already developed at the European scale by Melcher et al. (2007) and Schmutz et al. (2007b). However, their approach applied only to rivers belonging to FATs defined in their studies and there was need to extend the geographic range. Moreover, they used stepwise discriminant analysis to predict the fish types for impacted conditions, but according to Schmutz et al. (2007a), a disadvantage of discriminant function analysis is that the contributions of individual environmental variables are hidden in the discriminant functions of the model because of its multi-dimensional nature. In contrast, we searched for environmental variables that were able to predict the modelled river types for impacted conditions in a more traceable way.

### Metrics selection and pressure specific reaction

Finally, seventeen out of our 129 candidate metrics showed a significant response to specific and multiple pressures in four river types. Pont et al. (2007) defined 10 metrics that showed the best response to human pressures (slight vs. strong impact, but not pressure specific) for the European Fish Index (EFI): These were two metrics related to trophic structure (density of omnivorous species and density of insectivorous species), two metrics related to reproduction guilds (density of phytophilic species and relative abundance of lithophilic species) and two metrics related to physical habitat (number of benthic species and number of rheophilic species). Furthermore, relative number of tolerant and intolerant species reflected the capacity of fish assemblages to support disturbance in general, and two metrics reflected migratory species richness. The EFI+ project was another attempt to evaluate the ecological status of European rivers by one index (EFI+ Consortium 2009). The EFI+ consists of four final fish metrics, wherein two of the following metrics are selected, depending on a fish zone (salmonid or cyprinid): Rheophilic reproduction habitat species richness, oxygen depletion intolerant species abundance, lithophilic reproduction habitat species abundance and abundance of individuals <15 cm of habitat intolerant species. And finally, in their exercise to develop a predictive index of biotic integrity for aquatic-vertebrate assemblages of western U.S. streams, Pont et al. (2009) retained 5 metrics (2 vertebrate and bentic metrics, one index on assemblages’ tolerances and proportion of invertivore–piscivore species as well as proportion of lithophilic-reproducing species).

In contrast, our final selection did not contain migratory, insectivorous, benthivorous, lithophilic and rheophilic metrics, as we applied strict and standardised rules through the whole metric selection process to avoid redundancy (correlations >0.7) – many metrics therefore were removed in a stepwise procedure (see Annex Table 2, Annex Table 3, Annex Table 4, Annex Table 5). However, our selection also contained two habitat metrics responding to water quality-, hydromorphological- and to multiple pressures in HWS and LLR: density of rheopar species (HabSp_RHPAR_dens) and percent biomass of species tolerant to habitat degradation (HTOL_HTOL_perc_biom). For MES and LLR, our selection also contained three trophic level metrics that responded to hydromorphological- and multiple pressures: Percent piscivorous species (Atroph_PISC_perc_nsp), percent omnivorous species (Atroph_OMNI_perc_nsp) and percent biomass omnivorous species (Atroph_OMNI_perc_biom). One biodiversity metric (Nsp_all) responded to water quality-, hydromorphological- and to multiple pressures in HWS. This metric is generally expected to increase along the longitudinal course of a river but also to decrease with increasing environmental degradation in naturally poor species river types. In HWS, an increase of this metric therefore mainly shows a response that is described as a potamalisation-effect (Jungwirth et al., 1995, Schmutz et al., 2000).

Although Logez and Pont (2011) and partly the EFI+ project considered individual fish body size in order to distinguish between different life stages to detect human disturbances in European coldwater streams, we decided not to use metrics based on size classes/life stages, as the information on fish length was not available for most sites of our dataset and coverage of whole countries would have been lost. Hence, we tested this aspect indirectly by considering density- and biomass metrics with the hypothesis that responding biomass metrics tend to represent a reaction of adult- and density metrics of juvenile fish.

**Fig. 7:**
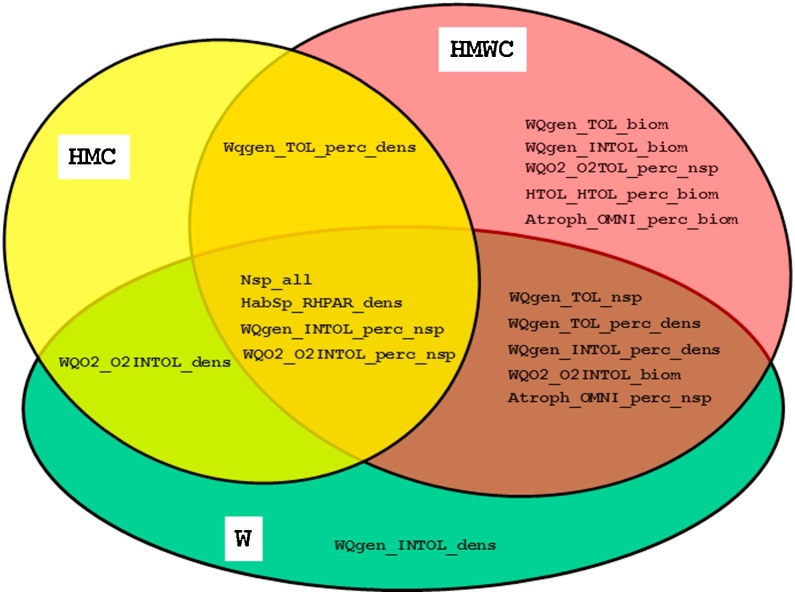
Venn diagram showing fish metrics with relevant response to specific and multiple pressures.

Moreover, we found differences between river types in terms of metrics response, i.e. we observed a shift from intolerant metrics in HWS to tolerant metrics in LLR. This is an obvious proof that various metrics are needed to show the response to pressures in different river types – an important aspect to be considered in further attempts for fish-based assessment on a wider geographical range. Furthermore, the seven “diagnostic metrics”, that showed a pressure specific reaction (exclusively to water quality or hydromorphological pressures) are only valid for three river types (HWS, LLR and MES). For MGR, no pressure specific metric was found (Table 6). Overall, metrics more often responded to multiple pressures than to specific pressures. Fig. 7 also shows that although some metrics are similar, they passed the redundancy tests and therefore are able to give additional information on e.g. the response of size-classes to pressures. Moreover, a clear shift from specific pressures to multiple pressures can be detected: The association between density-related metrics and specific pressures (either W or HMC) is almost total (only exception of *WQO2_O2Intol_biom*). However, in response to multiple pressures, there is a combination of biomass-related metrics and density-related metrics.

### Weaknesses and uncertainties

Our dataset covered a wide range of different ecoregions across Europe. Although the basis of our work was a common database provided by 14 countries, there were some inhomogeneities and a paucity of data for some areas (EFI+ Consortium, 2007). For example, in terms of characterisation of human pressures, Schinegger et al. (2012) has already shown that there are data gaps for particular regions of Europe (e.g. south-eastern countries) and in certain river types (particularly in large rivers) in the EFI+ dataset. Therefore, our pressure analysis was conducted on a general level–i.e. we focused on two specific pressures and their combinations. We are aware that the collective term “hydromorphological pressures” implies various single pressures that influence the response of certain metrics differently in terms of intensity and direction (see Schinegger et al., 2012 for details). However, to find metrics on a pan-European scale and for various river types, more accurate data are needed on a single pressure level for future attempts. Furthermore, we agree with Hering et al. (2010), who stated that other stressors such as land use, climate change, siltation, new toxic substances and alien species will be important for future work and that diagnostic metrics are currently only available for common types of degradation. Finally, we support the findings of Ormerod et al. (2010), that there are major challenges to understand the nature of multiple-stressor effects on species populations, communities and ecosystems, to identify and prioritise the major management issues and to seek the means to identify, diagnose and tackle multiple-stressors effects.

## Figures and Tables

**Annex Table 1:** Association of most frequent species in the dataset with various guilds: WQgen (Water quality general), WQO2 (Water quality O2 concentration), HTOL (Habitat degradation), Hab (Preferred living habitat), Atroph (Trophic situation), Mig (Migration), Repro (Reproduction habitat), HabSp (Spawning habit). Types: INTOL = intolerant, TOL = tolerant, IM = intermediate, RH = rheophilic, EURY = eurytopic, DETR = feeding on detritus, INSV = insectivorous, OMNI = omnivorous, PISC = piscivorous. RESID = resident, POTAD = potamodromous, LONG = long distance migrant. LITH = lithophilic, PELA = pelagophilic, SPEL = speleophilic, PSAM = psamnmophilic, PHLI = phyto-lithophilic.

Species	Family	WQgen	WQO2	HTOL	Hab	Atroph	Mig	Repro	HabSp
Alburnoides bipunctatus	Cyprinidae	INTOL	INTOL	INTOL	RH	INSV	RESID	LITH	RHPAR
Anguilla anguilla	Anguillidae	TOL	TOL	TOL	EURY	INSV	LONG	PELA	LIPAR
Barbatula barbatula	Nemacheilidae	IM	IM	IM	RH	INSV	RESID	LITH	EUPAR
Cottus gobio	Cottidae	INTOL	INTOL	INTOL	RH	INSV	RESID	SPEL	RHPAR
Gobio gobio	Cyprinidae	IM	INTOL	TOL	RH	INSV	RESID	PSAM	RHPAR
Leuciscus souffia	Cyprinidae	INTOL	INTOL	INTOL	RH	INSV	RESID	LITH	RHPAR
Phoxinus phoxinus	Cyprinidae	IM	INTOL	INTOL	RH	INSV	RESID	LITH	EUPAR
Pseudochondrostoma duriense	Cyprinidae	IM	O2IM	HIM	RH	DETR	POTAD	LITH	RHPAR
Rutilus rubilio	Cyprinidae	IM	IM	IM	EURY	INSV	RESID	PHLI	RHPAR
Rutilus rutilus	Cyprinidae	TOL	TOL	TOL	EURY	OMNI	POTAD	PHLI	EUPAR
Salmo salar	Salmonidae	INTOL	INTOL	INTOL	RH	PISC	LONG	LITH	RHPAR
Salmo trutta	Salmonidae	INTOL	INTOL	INTOL	RH	INSV	POTAD	LITH	RHPAR
Squalius pyrenaicus	Cyprinidae	IM	IM	IM	EURY	INSV	RESID	LITH	EUPAR

**Annex Table 2:** Pearson's correlation coefficient for metrics in HWS (before final selection), metrics in bold are finally selected. Values in bold indicate a correlation coefficient >0.7.

	Metrics HWS								
	WQO2_O2INTOL_biom	HabSp_RHPAR_dens	HTOL_HINTOL_dens	WQgen_INTOL_dens	WQO2_O2INTOL_dens	perc_nha_WQgen_INTOL	WQgen_INTOL_perc_nsp	Mig_POTAD_dens	Nsp_all
**WQO2_O2INTOL_biom**	**1.00**	0.49	0.43	0.65	0.44	0.43	0.30	0.52	−0.21
**HabSp_RHPAR_dens**	0.49	**1.00**	0.55	0.64	0.47	0.07	0.09	**0.77**	0.11
HTOL_HINTOL_dens	0.43	0.55	**1.00**	0.59	**0.95**	0.10	0.14	0.56	−0.01
**WQgen_INTOL_dens**	0.65	0.64	0.59	**1.00**	0.62	0.48	0.43	**0.75**	−0.26
**WQO2_O2INTOL_dens**	0.44	0.47	**0.95**	0.62	**1.00**	0.14	0.18	0.50	−0.01
perc_nha_WQgen_INTOL	0.43	0.07	0.10	0.48	0.14	**1.00**	**0.83**	0.23	−0.62
**WQgen_INTOL_perc_nsp**	0.30	0.09	0.14	0.43	0.18	**0.83**	**1.00**	0.22	−0.65
Mig_POTAD_dens	0.52	**0.77**	0.56	**0.75**	0.50	0.23	0.22	**1.00**	−0.10
**Nsp_all**	−0.21	0.11	−0.01	−0.26	−0.01	−0.62	−0.65	−0.10	**1.00**

**Annex Table 3:** Pearson's correlation coefficient for metrics in MGR (before final selection), metrics in bold are finally selected. Values in bold indicate a correlation coefficient >0.7.

	Metrics MGR					
	HTOL_HTOL_nsp	WQgen_TOL_nsp	HTOL_HTOL_perc_biom	HTOL_HTOL_perc_dens	WQgen_TOL_perc_dens	WQgen_TOL_perc_nsp
HTOL_HTOL_nsp	**1.00**	**0.96**	**0.73**	**0.72**	0.66	**0.76**
**WQgen_TOL_nsp**	**0.96**	**1.00**	0.67	0.69	0.66	**0.79**
HTOL_HTOL_perc_biom	**0.73**	0.67	**1.00**	**0.80**	**0.72**	**0.70**
HTOL_HTOL_perc_dens	**0.72**	0.69	**0.80**	**1.00**	**0.91**	**0.74**
**WQgen_TOL_perc_dens**	0.66	0.66	**0.72**	**0.91**	**1.00**	**0.74**
WQgen_TOL_perc_nsp	**0.76**	**0.79**	**0.70**	**0.74**	**0.74**	**1.00**

**Annex Table 4:** Pearson's correlation coefficient for metrics in LLR (before final selection), metrics in bold are finally selected. Values in bold indicate a correlation coefficient >0.7.

	Metrics LLR								
	WQgen_TOL_biom	WQO2_O2TOL_nsp	HTOL_HTOL_perc_biom	WQgen_INTOL_perc_biom	WQgen_TOL_perc_biom	WQgen_INTOL_perc_dens	Atroph_PISC_perc_nsp	WQO2_O2TOL_perc_nsp	Atroph_OMNI_perc_biom
**WQgen_TOL_biom**	**1.00**	0.53	0.41	−0.34	0.57	−0.22	0.01	0.36	0.46
WQO2_O2TOL_nsp	0.53	**1.00**	0.46	−0.41	0.46	−0.35	0.06	**0.74**	0.38
**HTOL_HTOL_perc_biom**	0.41	0.46	**1.00**	−0.88	**0.75**	−0.60	0.15	0.49	0.64
WQgen_INTOL_perc_biom	−0.34	−0.41	−0.88	**1.00**	−0.67	**0.71**	−0.06	−0.44	−0.66
WQgen_TOL_perc_biom	0.57	0.46	**0.75**	−0.67	**1.00**	−0.42	0.06	0.46	**0.84**
**WQgen_INTOL_perc_dens**	−0.22	−0.35	−0.60	**0.71**	−0.42	**1.00**	−0.06	−0.36	−0.45
**Atroph_PISC_perc_nsp**	0.01	0.06	0.15	−0.06	0.06	−0.06	**1.00**	−0.02	−0.01
**WQO2_O2TOL_perc_nsp**	0.36	**0.74**	0.49	−0.44	0.46	−0.36	−0.02	**1.00**	0.35
**Atroph_OMNI_perc_biom**	0.46	0.38	0.64	−0.66	**0.84**	−0.45	−0.01	0.35	**1.00**

**Annex Table 5:** Pearson's correlation coefficient for metrics in MES (before final selection), metrics in bold are finally selected. Values in bold indicate a correlation coefficient >0.7.

	Metrics MES															
	Atroph_INSV_biom	WQgen_INTOL_biom	WQO2_O2INTOL_biom	WQgen_INTOL_dens	HTOL_HTOL_nsp	WQgen_TOL_nsp	WQgen_INTOL_perc_biom	HTOL_HINTOL_perc_dens	WQgen_INTOL_perc_dens	WQgen_TOL_perc_dens	WQO2_O2INTOL_dens	WQgen_INTOL_perc_nsp	WQO2_O2INTOL_perc_nsp	Atroph_OMNI_nsp	Atroph_OMNI_perc_dens	Atroph_OMNI_perc_nsp
Atroph_INSV_biom	**1.00**	**0.92**	**0.77**	0.64	0.28	0.10	0.38	0.42	0.41	−0.23	0.46	0.38	0.37	−0.02	−0.28	−0.18
**WQgen_INTOL_biom**	**0.92**	**1.00**	**0.77**	0.65	0.22	0.07	0.52	0.44	0.50	−0.23	0.46	0.42	0.37	−0.05	−0.26	−0.18
WQO2_O2INTOL_biom	**0.77**	**0.77**	**1.00**	**0.76**	−0.04	−0.17	0.54	0.56	0.58	−0.36	0.62	0.53	0.51	−0.20	−0.32	−0.26
**WQgen_INTOL_dens**	0.64	0.65	**0.76**	**1.00**	0.00	−0.10	0.50	0.56	0.67	−0.30	0.60	0.52	0.46	−0.16	−0.28	−0.27
HTOL_HTOL_nsp	0.28	0.22	−0.04	0.00	**1.00**	**0.89**	−0.15	−0.20	−0.18	0.31	−0.20	−0.23	−0.25	**0.78**	0.26	0.34
**WQgen_TOL_nsp**	0.10	0.07	−0.17	−0.10	**0.89**	**1.00**	−0.29	−0.32	−0.26	0.52	−0.34	−0.34	−0.40	**0.83**	0.43	0.49
WQgen_INTOL_perc_biom	0.38	0.52	0.54	0.50	−0.15	−0.29	**1.00**	**0.75**	**0.79**	−0.41	**0.79**	**0.76**	**0.73**	−0.36	−0.41	−0.38
HTOL_HINTOL_perc_dens	0.42	0.44	0.56	0.56	−0.20	−0.32	**0.75**	**1.00**	**0.75**	−0.46	**0.91**	**0.70**	**0.79**	−0.41	−0.53	−0.51
**WQgen_INTOL_perc_dens**	0.41	0.50	0.58	0.67	−0.18	−0.26	**0.79**	**0.75**	**1.00**	−0.40	**0.80**	**0.79**	0.62	−0.38	−0.42	−0.43
**WQgen_TOL_perc_dens**	−0.23	−0.23	−0.36	−0.30	0.31	0.52	−0.41	−0.46	−0.40	**1.00**	−0.51	−0.37	−0.45	0.41	0.69	0.51
**WQO2_O2INTOL_dens**	0.46	0.46	0.62	0.60	−0.20	−0.34	**0.79**	**0.91**	**0.80**	−0.51	**1.00**	**0.77**	**0.83**	−0.42	−0.52	−0.50
WQgen_INTOL_perc_nsp	0.38	0.42	0.53	0.52	−0.23	−0.34	**0.76**	**0.70**	**0.79**	−0.37	**0.77**	**1.00**	**0.84**	−0.40	−0.41	-0.49
**WQO2_O2INTOL_perc_nsp**	0.37	0.37	0.51	0.46	−0.25	−0.40	**0.73**	**0.79**	0.62	−0.45	**0.83**	**0.84**	**1.00**	−0.43	−0.45	−0.52
Atroph_OMNI_nsp	−0.02	−0.05	−0.20	−0.16	**0.78**	**0.83**	−0.36	−0.41	−0.38	0.41	−0.42	−0.40	−0.43	**1.00**	0.57	**0.74**
Atroph_OMNI_perc_dens	−0.28	−0.26	−0.32	−0.28	0.26	0.43	−0.41	−0.53	−0.42	0.69	−0.52	−0.41	−0.45	0.57	**1.00**	**0.75**
**Atroph_OMNI_perc_nsp**	−0.18	−0.18	−0.26	−0.27	0.34	0.49	−0.38	−0.51	−0.43	0.51	−0.50	−0.49	−0.52	**0.74**	**0.75**	**1.00**
